# Bioequivalence of micronutrient powders to Corn-soy Blend on serum zinc concentration of children (6–36 months) with Moderate Acute Malnutrition in Thika urban slums, Kenya: A cluster-randomized controlled trial

**DOI:** 10.1371/journal.pone.0274870

**Published:** 2022-09-19

**Authors:** Juliana Kiio, Sophie Ochola, Ruth Nduati, Elizabeth Kuria, Scholastica Mathenge, Judith Okoth

**Affiliations:** 1 Dept. of Food, Nutrition & Dietetics, Kenyatta University, Nairobi, Kenya; 2 Dept. of Paediatrics & Child Health, University of Nairobi, Nairobi, Kenya; 3 Dept. of Medical laboratory Science, Kenyatta University, Nairobi, Kenya; 4 Dept. of Food Science & Technology, Jomo Kenyatta University of Agriculture and Technology, Juja, Kenya; Public Library of Science, UNITED KINGDOM

## Abstract

Zinc deficiency is common among children with Moderate Acute Malnutrition (MAM) and contributes to growth failure, increased morbidity and mortality. Diarrhoea and poor dietary practices are the main causes of zinc deficiency. Corn-soy Blend (CSB), the standard product in management of children with MAM has a limitation of poor micronutrient bioavailability. Micronutrient powders (MNPs) which are added at the point of consumption have a potential in improving micronutrient status however, scientific evidence on efficacy on improving the zinc status is scarce. A cluster-randomized clinical trial was designed to establish bioequivalence of MNPs to CSB on serum zinc status among children (6–36 months) with MAM in Thika informal settlements, Kenya. Sample size was calculated to show bioequivalence within ±20% limit. Twelve villages were randomized to four study groups. Three experimental groups received different formulations of MNPs added to unfortified CSB porridge as; multiple micronutrients containing zinc (CSB-MNP-A n = 84), multiple micronutrients without zinc (CSB-MNP-B n = 88) and zinc only (CSB-MNP-C n = 94). Control group (n = 80) received standard CSB fortified with multiple micronutrients. Standard amount of CSB was consumed in feeding centres for six months. Serum zinc concentration was assessed pre- and post-intervention. Data was analyzed based on treatment assignment regardless of adherence and drop-out status. Mixed effects linear regression was used to model pre-post change in serum zinc concentration, adjusting for clustering effect and baseline differences. Bioequivalence was assessed using two one-sided t-tests. At baseline, 84.4% were zinc deficient (serum zinc <65μg/dL) and zinc intake was sub-optimal (<3 mg/day) for 95.7% of children. Mean change in serum zinc concentration was significantly higher (p = 0.024) in CSB-MNP-A (18.7 ± 2.1) *μ*g/dL compared to control group (11.8 ± 2.6 *μ*g/dL). MNPs are not bioequivalent to CSB within the ±20% bioequivalence limit. MNPs are more effective in improving serum zinc status compared to CSB. Trials with larger sample sizes are recommended to validate the current findings.

**Trial registration**: Pan African Clinical Trials Registry: PACTR201907492232376.

## Introduction

Globally, acute malnutrition is a threat to child survival and is attributed to nearly half of deaths among children [1]. Three quarters of the children have Moderate Acute Malnutrition (MAM) [2]. MAM children (MUAC≥115 mm to <125 mm or W/H between ≥ - 3 and <-2 Z-score [3] are hooked into the vicious cycle of recurring sickness and growth faltering which increases their risk of becoming severely malnourished, an immediately life threatening condition [4]. In Kenya, the prevalence of acute malnutrition among urban slums is high (6.3%) [5, 6] and increasing mortality trends have been reported [7]. The high slum population warrants the management of children with acute malnutrition to be made a public health priority.

Micronutrient deficiencies are common among MAM children and usually co-exist [8]. Zinc deficiency is more prevalent among children with acute malnutrition compared to the apparently healthy counterparts [9]. In Kenya, information on zinc deficiency among MAM children in lacking. However, according to the Kenya micronutrient survey conducted in 2011, 83.3% children aged below 59 months have zinc deficiency [10]. Zinc deficiency contributes to the burden of disease and is associated with an increased risk of mortality from diarrhoea, pneumonia and malaria by 13–21% [11, 12]. Diarrhoeal morbidity further exacerbates zinc deficiency due to gastro-intestinal losses of the micronutrient [13]. Acute and persistent diarrhoea which is rampant among children living in urban informal settlements due to poor sanitary conditions increases their vulnerability to zinc deficiency. Increased demand for zinc due to rapid growth [14] coupled with sub-optimal feeding practices in resource-poor settings [15] as well as diarrheal morbidity pre-disposes children aged 6–36 months to zinc deficiency [16]. Typical diets in developing countries mainly consist of starchy staples; cereals, tubers and legumes which have low level of bio-available zinc. The foods richest in zinc are from animal sources, but these are not often included in children diets under resource poor settings because of limited availability, affordability, entrenched custom or religion. Plant sources of zinc contain high levels of phytic acid, a potent inhibitor of zinc absorption [17, 18]. As a result, the amount of zinc available for absorption from such diets is low and probably the primary cause of zinc deficiency.

Nutritional management of children with MAM is based on provision of supplementary food products such as fortified blended foods [19]. The management aims at meeting protein and energy as well as multiple micronutrient requirements [20]. Corn-soy Blend (CSB) is the standard food for rehabilitation of children with MAM [19]. A major limitation of CSB however, is the poor bioavailability of micronutrients due to its high level of anti-nutrient content [21]. **The anti-nutrients** form complexes with zinc, iron and other minerals leading to poor bioavailability [22]. The poor bioavailability has driven researchers to finding alternative supplementary products for managing children with MAM. Home fortification of supplementary food products at the point of consumption using Micronutrient Powders (MNPs) may be a better approach of improving the micronutrient bioavailability possibly due to reduced matrix effects compared to CSB. The current study was therefore designed to investigate the bioequivalence of MNPs compared to CSB in improving serum zinc concentrations among children with MAM aged 6–36 months.

## Materials and methods

### Study design

The study was a 4-parallel arm cluster randomized controlled trial conducted from April to September, 2013 involving three treatment groups and a control with an allocation ratio of 1:1:1:1. It was designed to test for the bioequivalence of MNP fortification compared to the CSB on the serum zinc status of children with MAM. The study was conducted in Kiandutu and adjacent slum areas in Thika West District, Kiambu County which is approximately 40 km north of the City of Nairobi. Kiandutu is one of the largest informal settlements located outside Nairobi, the capital city of Kenya. The study area consists of twelve villages; Mtatu, Biashara, Muslim, Kianjau, Molo, Mkira, Wahome, Athena settlement, Makongeni, Umoja, Gachagi and Madharau. The informal settlements are characterized by low socio-economic status, poor sanitation, food insecurity and high levels of child malnutrition [23]. Children with MAM aged 6–36 months (MUAC≥ 115 mm to <125 mm or W/H between ≥ - 3 and <-2 Z-score) without bilateral oedema were eligible. A sample size large enough to detect a bioequivalence within ±20% rule with 80% power and 5% significance level [24] using two one-sided tests of equivalence was determined based on outcome of a pilot study conducted in Kiang’ombe slums. Considering serum zinc concentration of children in the treatment group (CSB-MNP-A) 76.4 *±18*.*2μ*g/dL and control group (CSB) 68.4 *±5*.*5μ*g/dL yielded a Cohen’s *d* [25] “medium” effect size of 0.595. A *design effect of* 1.3 and a projected 20% attrition yielded a sample size of 78 per study group.

### Food supplementation products and study groups

Corn-soy Blend (80:20) prepared using World Food Programme formulation [19], cooking oil and MNPs were sourced from East Africa Nutraceuticals Kenya Limited, Bidco Oil Refineries Limited, Kenya and Hexagon PVT, India respectively. White refined sugar purchased locally. CSB was supplied in 50-Kilogram bags and MNPs supplied as Sprinkles^tm^ packaged in thirty one-gram sachets. Sugar and oil were pre-mixed aseptically with CSB flour in a central place every two weeks, re-packaged in plastic bags, heat sealed and distributed according to the requirements of the different feeding centres. The pre-mix contained CSB (50 g), vegetable oil (5 g) and sugar (8.75 g) of added to taste. Each child consumed 63.75g of the CSB mixture in the form of one standard cup of porridge once daily in designated feeding centres for a period of six months. Consumption of the standard cup of CSB provided an extra 237.7 Kcal and 6.9 g of protein, with 29% of the energy coming from the added oil.

The control group consumed daily CSB containing the same level of multiple micronutrients as the MNPs. For the experimental groups, the MNP mix was sprinkled into a standard preparation of unfortified CSB porridge. Children in the CSB- MNP-A consumed daily multiple micronutrients containing zinc whereas those in the CSB-MNP-B group consumed the multiple micronutrients mix with no added zinc. However, children in the CSB-MNP-C group consumed daily MNP containing zinc only. The micronutrient fortification level of the intervention products is summarized in Table 1.

**Table 1:** The micronutrient fortification levels of the intervention products.

Micronutrient	CSB-MNP-A	CSB-MNP-B	CSB-MNP-C	CSB
Vit A (IU)	1250	1250	-	1250
Vit C (mg)	30	30	-	30
Thiamine (mg)	0.5	0.5	-	0.1
Riboflavin (mg)	0.5	0.5	-	0.4
Niacin (mg)	6	6	-	5
Folic Acid (mcg)	160	160	-	50
Vit. B_12_ (mcg)	0.9	0.9	-	1
Calcium (mg)	-	-	-	100
Iron (mg)	12.5	12.5	-	8
Zinc (mg)	5	-	5	5

### Recruitment and enrolment of study participants

Potential study participants were screened from the nearby community health centres as well as door-to-door screening conducted by Community Health Volunteers (CHVs). The nutrition status of the referred children was verified by research staff using MUAC and weight-for-height measurements and clinical examinations conducted by a clinical officer. Children aged 6–36 months (MUAC≥ 115 mm to <125 mm or W/H between ≥ - 3 and <-2 Z-score) without bilateral oedema were eligible. Eligible children whose parents/caregivers consented to participate, had the intention of staying in the area during the study period and were willing to be visited in their homes and had been residents in the area for at least 6 months prior to the study were recruited. Children consuming fortified foods or on micronutrient supplementation programs (except routine Vit. A supplementation) or those enrolled to other food supplementation programs were excluded. Sick children were treated at the health centre whereas chronically ill (as verified from health cards) and severely malnourished (W/H<-3SD or MUAC<115 mm) or anaemic (Hb<7.0 g/dl) children were referred to seek further medical attention and excluded from the study.

During enrolment, mothers were invited to a central point in each village/cluster for a detailed introduction to the study aims and procedures without disclosing the assigned treatments and study hypotheses by the researcher and assistants.

### Feeding centres and intervention monitoring

Feeding centres were established taking into account ease of access by mothers and state of hygiene including availability of toilets and potable running water. Cooking utensils, standard cups, soap and charcoal was provided. Demonstration charts on preparation of the porridge and addition of MNPs were developed and displayed in the feeding centres and the training conducted by the research team. Porridge was prepared centrally and mothers requested to promptly bring their children early in the morning before the child was fed. For poor feeders, mothers were advised to feed their children first on a small proportion of the porridge mixed with the full contents (1g) of the MNPs and subsequently the remaining amount of the porridge. For the few children attending informal daycare centres, the porridge was delivered and feeding monitored closely by the research assistants. At enrolment, all study children aged above one year were de-wormed at the start of intervention using Mebendazole (500 mg).

Quality spot checks were carried out weekly by the research team at the feeding centres to monitor the standard procedures of hygiene, porridge preparation and feeding of children. To monitor compliance, daily records on quantities cooked, attendance and consumption of the porridge were kept. Cases of pouring and vomiting were recorded and considered as a day’s non-compliance. The total number of intervention days was 168 days. Adverse effects of the treatments were investigated by asking mothers of any possible unexpected responses of the children which they perceived to be associated with the current intervention treatments such as allergic reactions, vomiting and diarrhoea. The mothers were encouraged to report the adverse effects to the research assistants who would immediately contact the researcher. The researcher would interrogate the mothers and with the assistance of a clinician, an assessment on the cause, severity and seriousness was done. If the adverse effect was not serious it was recorded and close monitoring done. Serious adverse effects such as those with severe symptoms, requiring hospitalization or death during the intervention period were however not reported.

### Data collection procedures

The households of the eligible participants were visited by the trained enumerators. After obtaining informed consent, primary caregivers to the index child was interviewed to collect baseline data on the socio-demographic characteristics of the index child and their family, morbidity and the feeding patterns. Demographic and social economic characteristics of the household were assessed using a standard questionnaire [26]. Morbidity was assessed using a two-week morbidity recall questionnaire. A 24-hour recall questionnaire was used to assess nutrient intake for two non-consecutive days which were randomly chosen. Primary caregivers were asked to give a full description of ingredients in mixed foods, cooking method and the amounts eaten were estimated using household measures of cups, spoons, bowls, plates, match boxes and food models. Forgotten foods, snacks, drinks and street foods were probed and information on whether the consumption was usual was collected. Morbidity and dietary assessment was done monthly.

Physical examination to check for bilateral oedema and anthropometric measurements were carried out at the feeding centres on a monthly basis by the enumerators. SECA electronic weighing scale to the nearest 0.1 kg was used to weigh children who were near nude (light clothing and without shoes). Calibration was done before every weighing to a zero reading. A stadiometre (UNICEF) with an accuracy of up to 0.1 cm was used to measure height/length. Recumbent length was taken for children aged less than two years or those whose length was shorter than 87 cm and height for those whose length was greater than 87cm. Birth date was verified from Health Cards for most children apart from a few children whose age was estimated using either a localized calendar of events or another child of a similar age in the household or in the close proximity, whose date of birth was known or recorded.

Serum zinc concentration, serum albumin and C-Reactive Proteins (CRP) were assessed pre- and post-intervention. Non-fasting morning blood samples (5mls) were drawn by a phlebotomist using venipuncture using 23-gauge butterfly needles; placed into a clot tube (BD Vacutainer^®^ - red top). A study limitation was that trace-element-free vacutainers were not used. After 30 minutes, the samples were centrifuged (1000 *g*) for 15 min, aliquots of serum was transferred into coded trace-element free cryovials, wrapped in aluminium foil and put in a cool box containing ice packs and transported to the KEMRI Centre for Public Health Research (CPHR) laboratory transferred to a freezer (-20° C) till analysis. Screening for malaria using rapid diagnostic techniques and preparation of thick and thin smears were carried out by the health centre laboratory technicians. Serum zinc concentration was determined using Atomic Absorption Spectrophotometer (Shimadzu Corporation, Kyoto, Japan, AA-6200). Validity of the method was ensured using standard reference material for zinc determination. Inter-laboratory, inter- and intra-sample testing was also done. During the analysis, zinc standards were read after every 10 samples and re-calibration done if any change in standard readings was noticed. Zinc deficiency was defined as morning non-fasting serum zinc concentration of <65μg/dL [27].

CRP concentration was determined using immuno-turbidimetric method using fully automated chemistry analyzer HUMASTAR 600 (Hitachi, Japan). The analytical reagents were as follows: Reagent 1- CRP buffer (pH 7.5) containing polyethylene glycol (20mmol/L) (Sigma-Aldrich, UK) and sodium azide (0.095%) (Sigma-Aldrich, UK). Reagent 2- CRP antiserum containing monospecific goat anti-human CRP antibodies (Invitrogen PA1-18303) at 1:10, 000 dilution. The CRP standard was stabilized human serum certified reference material 470 (US RPPHS lot 91/0619). Absorbance was read at 340nm and the concentration determined from calibration curves. Re-calibration was done before every run. Elevated acute phase protein was defined as CRP concentration > 5 mg/L and the BRINDA regression correction method used to adjust serum zinc concentrations for markers of inflammation [28]. Serum albumin concentration was determined by Doumas, Watson and Biggs method [29], using Fortress diagnostic® albumin reagent (Fortess Diagnositics, UK). Dilution of the serum was prepared by taking 250*μ*l of serum and adding 4.75ml of double distilled water. After vortexing, 1 ml of the solution of bromcresol green reagent was added, vortexed and left for 5 minutes at room temperature. Within each assay, all samples were analyzed using the same reagents. The albumin standard given in the kit was used to obtain the standard absorbance. Absorbance was read at 615*μ*m using a Chemical Analyzer (HUMASTAR 600—Hitachi, Japan). Normal serum albumin concentration among children <3 years of 2.9–5.8g/dl was considered [30].

The trial was registered retrospectively. This was mainly because the author had limited prior knowledge on trial registration. The authors confirm that this did not in any way influence the reporting of the findings and the research methodologies. The authors confirm that all ongoing and related trials for this intervention are registered.

### Ethical considerations

Ethical approval was obtained from the Ethical Review Committee (ERC) of the Kenya Medical Research Institute (KEMRI)–NON SSC PROTOCOL NO. 281. Informed written consent was obtained from the mothers/primary caregivers of the children. Anonymity and confidentiality was ensured and the participants were identified using codes. There were no risks or harms associated with the intervention.

The complete study protocol can be accessed in S1 Protocol.

### Sample size calculation and data analysis

The sample size per study group was calculated using findings of the pilot study as follows:

$$Nadjusted=\frac{(Z\alpha +Z\beta {)}^{2}CV^{2}}{(\partial -|\in |{)}^{2}}=(1.64+1.28{)}^{2}0.02^{2}(0.02-0.117{)}^{2}=50$$ (1)

Where;

$$CV=\sqrt{MSE}/\mu ref=\sqrt{2.125}/68.4=0.02$$ (2)

$$\in =\frac{\mu t-\mu ref}{\mu ref}=\frac{76.4-68.4}{68.4}=0.117$$ (3)

N_unadjusted_: Number of study participants per group before adjustment of clustering effect, N_adjusted_: Number of study subjects per group after adjustment of clustering effect, CV: Coefficient of variation (Pilot study), δ:Bioequivalence limit (±20% considering bioequivalence rule), ε:Absolute values of mean differences among the study groups, Y: Standard normal deviate (95 probability error) is Z_α_ and Z_β_, MSE: Mean squared error, *μ*_*ref*_: Mean of the reference group, *μ*_*T*_: Mean of treatment group.

Design effect (DEFF) was calculated [31] using an estimated average cluster size of 20 since population statistics at the village level were not available.


DEFF=1+(M−1)ICC=1+(20−1)0.018=1.3 (4)

$$Where;\;ICC=\frac{\left\{BMS-WMS\right\}}{\left\{BMS+(M-1)WMS\right\}}=\frac{\left\{1.2-2.2\right\}}{\left\{1.2(20-1)19\right\}}=0.018$$ (5)

DEFF: Design effect, ICC: Intra-cluster correlation coefficient, BMS: between cluster mean square, ANOVA (Pilot study), WMS: within cluster mean square, ANOVA (Pilot study), M: Cluster size

Data was cleaned, coded and entered into EpiData version 3.1, further cleaned in EXCEL (2007) and exported to STATA version 13 for statistical analysis. Socio-economic status (SES) score was constructed using Principal Components Analysis (PCA) considering the housing characteristics (wall, floor and roofing), land ownership, possession of household goods, bank account and cooking fuel [26]. Factor analysis was done using a correlation matrix and the principal components extracted based on eigenvalues >1. Varimax rotation yielded factor scores for each characteristic or asset. A total score of the individual household was obtained and the households categorized into three wealth quintiles; least poor, medium poor and poorest. The 24-hr recall data was entered into Nutri-survey (2007) program and exported to Excel (2007). ENA for SMART was used to analyze anthropometric data. Anthropometric status was evaluated using WHO growth standards [3]. Normality of data was checked using QQ plots and Histograms.

All the randomized participants were included in the data analysis based on treatment assignment regardless of adherence and drop-out status. T-test and Mann-Whitney U test were used to compare the means of normally and non-normally distributed continuous variables respectively. For categorical data, chi-square test and Fisher’s exact test (N<5) was used. Intervention groups were compared to the control group to demonstrate the success of randomization. To check for attrition bias, children who dropped out of the study were compared to those completing the study. Missing at random was assumed as the study drop out was not in any way associated with the intervention. To determine the effect of the intervention, each of the experimental groups was compared to the control group using two-sample t-test (two-tailed). Mixed effects linear regression was used to model the pre-post intervention changes in serum zinc concentration of the study groups, adjusting for clustering effect as well as the age, sex and SES of the children. Average bioequivalence of the MNPs to CSB was concluded if the confidence interval (95% CI -0.2*control, +0.2*control) of the pre-post intervention difference in mean serum zinc concentration for the two formulations was within the bioequivalence limit (±20%) using two one-sided t-tests (TOST). Statistical significance was determined at 95% level of confidence (two-tailed) at p< 0.05. Missing data values of serum zinc were imputed using multiple imputations with five itinerations and pooled estimates used in the final analysis. The multiple imputation model included baseline and post intervention values, treatment group, sex and age.

## Results

### Study profile

**Fig 1:**
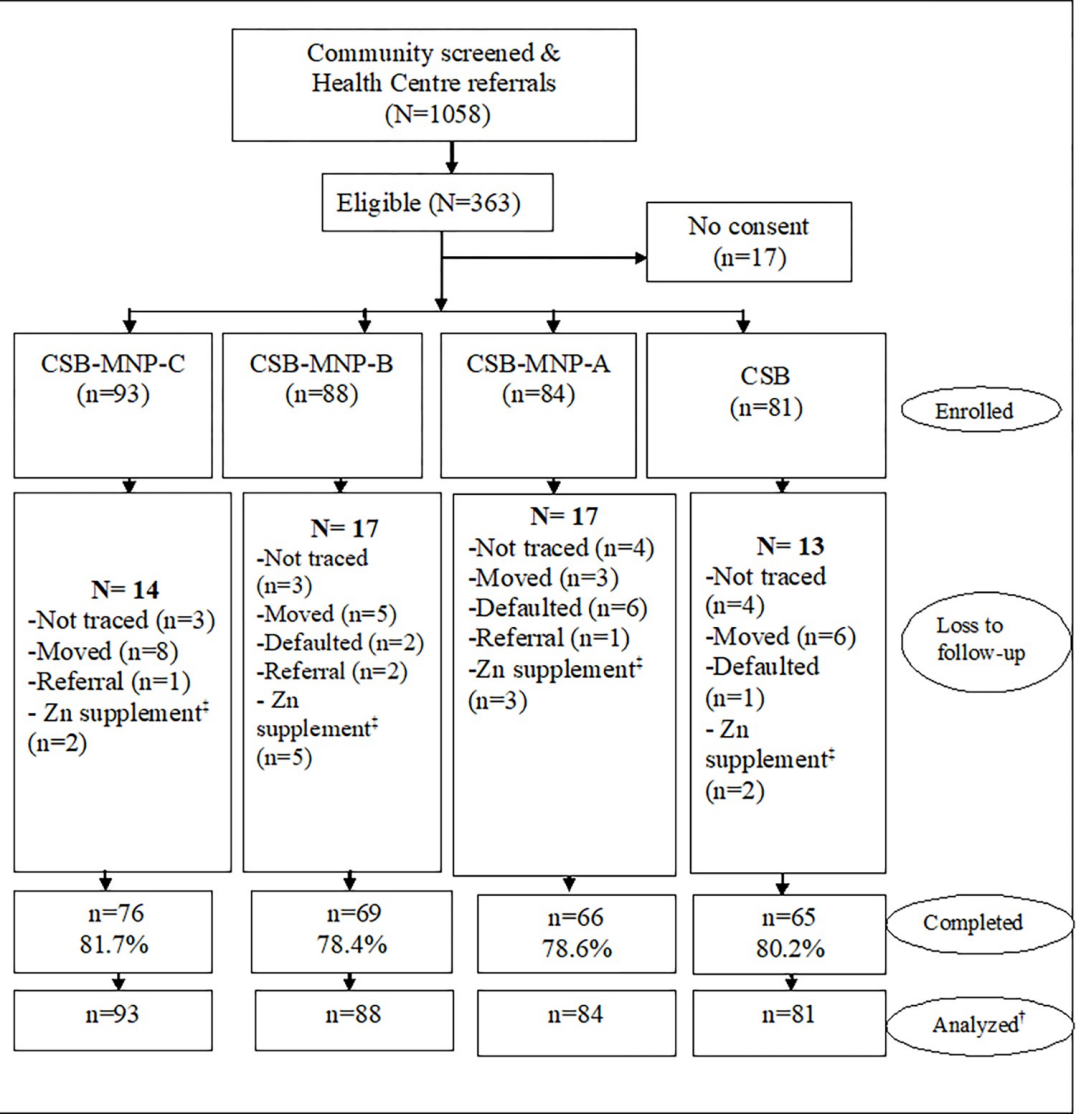
Trial profile. ^‡^Received therapeutic zinc dose from health facility; ^†^All participants were analyzed based on treatment assignment regardless of adherence and drop-out status.

Out of the total of 1058 children screened, 363 children met the eligibility criteria. However, mothers to 17 children did not consent to participate mainly as a result of unwillingness to have a blood sample drawn for the assessment. A total of 346 children were enrolled into the study; CSB- MNP-C (n = 94), CSB- MNP-B (n = 88), CSB-MNP-A (n = 84) and Control- CSB (n = 80). The completion rates of the participants was 81.7% (n = 76), 78.4% (n = 69), 78.6% (n = 66) and 80.2% (n = 65) in the study groups respectively, (20.2%) were lost to follow-up mainly due to relocation from the study area, non-traceability, defaulters and referral cases. Defaulters were considered as those not consuming the intervention products and/or attending in less than 80% of the intervention days. Therapeutic zinc supplementation from the health facility during the study period was given to twelve children and hence they were not included in the analysis. Blood samples for nine, children were either absent or inadequate for serum analysis. All the children who were randomized to the study groups at baseline were included in the analysis of the outcomes using intention-to-treat whereby study participants were analyzed based on treatment assignment regardless of adherence and drop-out status. Fig 1 shows the trial profile.

### Baseline characteristics of study participants

The mean age of the children enrolled into the study was 19.4±8.3 months. Nearly half of the study children were girls (48.6%) and the mean age was 19.4±8.3, months. Most of mothers (79.5%) had attained primary education with only 20.5% having attained secondary school education. Mothers were mainly (65.9%) housewives whereas 34.1% obtained their income from small-scale businesses or casual employment. Paternal income was mainly (68.2%) from casual labour and small businesses and 31.9% of the fathers did not have formal employment. The study groups were similar in all the characteristics except for the SES and child age. At baseline, majority (84.4%) were zinc deficient (serum zinc concentration <65μg/dL) and zinc intake was sub-optimal (<3 mg/day) for 95.7%. Children in the CSB-MNP-C group were significantly older (p = 0.004) than children in the control group. Children in the CSB-MNP-A group had a significantly different SES status (p = 0.001) compared to the control group (Table 2). Baseline differences were controlled for in the analysis of the effects of the treatments. Comparison of children who completed the study and those who dropped out showed no significant differences on baseline characteristics (p> 0.050). There was no significant difference between study groups on the loss to follow-up (p = 0.438).

**Table 2:** Comparison of study groups at baseline.

	CSB-MNP-A (n = 84)	CSB-MNP-B (n = 88)	CSB-MNP-C (n = 94)	CSB (n = 80)
**Mothers Education levels**				
Primary and less (%)	66 (78.6)	67 (76.1)	72 (76.6)	70 (87.5)
Post-primary (%)	18 (21.4)	21 (23.9)	22 (23.4)	10 (12.5)
**Mothers Occupation**				
Unemployed /housewives (%)	54(64.3)	52 (59.1)	68 (72.3)	54 (67.5)
Employed/Small business (%)	30 (35.7)	36 (40.9)	26 (27.7)	26 (32.5)
**Paternal Occupation**				
Unemployed (%)	17 (27.9)	20 (32.3)	20 (29.9)	22 (37.9)
Employed/Small business (%)	44 (72.1)	42 (67.7)	47 (70.2)	36 (62.1)
**Household Characteristics**				
Household size, mean (SD)	4.5 (1.4)	4.7 (1.5)	4.9 (1.5)	4.9 (1.6)
SES, quintiles: Poorest (%)	28 (33.3)^***^	25 (28.4)	35 (37.2)	28 (35.0)
Middle poor (%)	19 (22.6)	31 (35.2)	30 (31.9)	36 (45.0)
Least poor (%)	37 (44.1)	32 (36.4)	29 (30.9)	16 (20.0)
**Childs Characteristics**				
Gender, Girl (%)	37 (44.1)	48 (54.6)	44 (46.8)	39 (48.8)
Age (months), mean (SD)	18.6 (7.5)	17.6(7.7)	22.3(8.8)^**^	18.7(8.6)
Dietary zinc (mg/d), mean (SD)	1.6(2.1)	1.1(0.7)	1.6(0.9)	1.1(0.6)
WHZ score, mean (SD)	-1.52(0.76)	-1.47(0.76)	-1.74(0.69)	-1.59(0.73)
MUAC (cm), mean (SD)	12.3 (0.28)	12.3 (0.28)	12.3 (0.31)	12.3 (0.27)
^†^Serum zinc *μg/dL*, median (P25, P75)	41 (44.0, 65.5)	43 (39.5, 61.5)	40 (39.6, 63.9)	34 (34.9, 61.6)
Serum albumin *g/dL*, mean (SD)	4.35 (0.83)	4.10 (0.98)	4.23 (1.01)	4.31 (0.99)
CRP *mg/L*, mean (SD)	12.8 (10.5)	13.0 (7.2)	14.1 (16.0)	12.2 (10.2)
Positive malaria smears n (%)	1 (1.2)	0 (0)	0 (0)	1 (1.3)
Diarrhoea (%)	39 (46.4)	45 (51.1)	41 (43.6)	32 (40.0)

^†^Adjusted for elevated CRP, **p<0.010, ***p <0.001 significant differences between treatment groups compared to the control group (t-test, Mann-whitney U-test and χ^2^), P25 = 25^th^ percentiles, P75 = 75^th^ percentiles

### Adherence and zinc intake during the intervention

The overall mean compliance days for the children who completed the study were 152.2±9.1 days. The total mean days complied for CSB- MNP-A, CSB-MNP-B, CSB-MNP-C and CSB was 150.8±9.9, 152.2±9.5, 152.9±8.5 and 153.1±9.1 respectively. Adherence level of ≥80% of the intervention days is considered adequate [32]. Compliance ranged from 89.7% to 91.1% among the study population with no significant difference between study groups (χ^2^-test, p = 0.917). During the intervention, all children in the study groups met the recommended RNI of zinc intake (<3 mg/day) apart from the CSB-MNP-B which had significant lower proportions (33.4%) of children meeting the RNI compared to the control group (χ^2^ -test p <0.001). However, phytate intake was not assessed.

### Bioequivalence of MNPs to CSB on serum zinc concentration

Compared to the control group, children in the CSB-MNP-A had significantly higher mean serum zinc concentration (t-test p = 0.001) whereas the levels were significantly lower among children in the CSB-MNP-B (t-test p = 0.001). No significant difference was observed between the control group and CSB-MNP-C (t-test p = 0.600) (Table 3).

**Table 3:** Post-intervention serum zinc, albumin and CRP concentration by study groups.

	CSB-MNP-A (n = 84)	CSB-MNP-B (n = 88)	CSB-MNP-C (n = 94)	CSB (n = 80)
Crude mean serum zinc, *μ*g/dL (SE) ^‡^	87.7 (2.0)***	59.7 (1.7)***	75.4 (1.5)	75.9 (1.9)
^*†*^Adjusted mean serum zinc *μ*g/dL (SE) ^‡^	66.4 (2.4)***	41.7 (2.5)***	54.3 (2.3)	56.8 (3.2)
^†^Pre-post change in serum zinc *μ*g/dL (SE)^¥^	18.7(2.1)*	-4.9(2.6)***	9.4(2.5)	11.8(2.6)
Serum alb *g/dL*, mean (SD)^‡^	4.3(0.9)	4.2(0.8)*	4.4(0.9)	4.6(1.0)
Hypoalbuminaemia, n (%) ^ǂ^	0(0)	2(2.9)	3(4.0)	1(1.5)
CRP mg/L, mean (SD)^‡^	9.8(4.8)	11.6(7.2)	9.3(6.6)	10.1(6.9)

*p <0.050

***p <0.001 Significant differences of individual treatment groups compared to the control group

^‡^ *t-test;* ^ǂ^*χ^2^;*

^¥^Mixed effects linear regression, adjusting for baseline serum concentration, clustering effect, age, sex and SES.

^†^Corrected for elevated CRP concentration; adjusted for age, sex and SES, clustering and baseline serum zinc concentration.

There was an increase in the mean serum zinc concentration for the CSB-MNP-A, CSB-MNP-C and CSB. However, mean serum zinc concentration of the CSB-MNP-B decreased. Compared to the control group, the pre-post change in serum zinc concentration was significantly higher for CSB-MNP-A (p = 0.024). Although the magnitude of change in the serum zinc concentration of the children in CSB-MNP-C was lower compared to the control group, no significant differences were observed between the two groups (p = 0.512) (Table 3).

**Fig 2:**
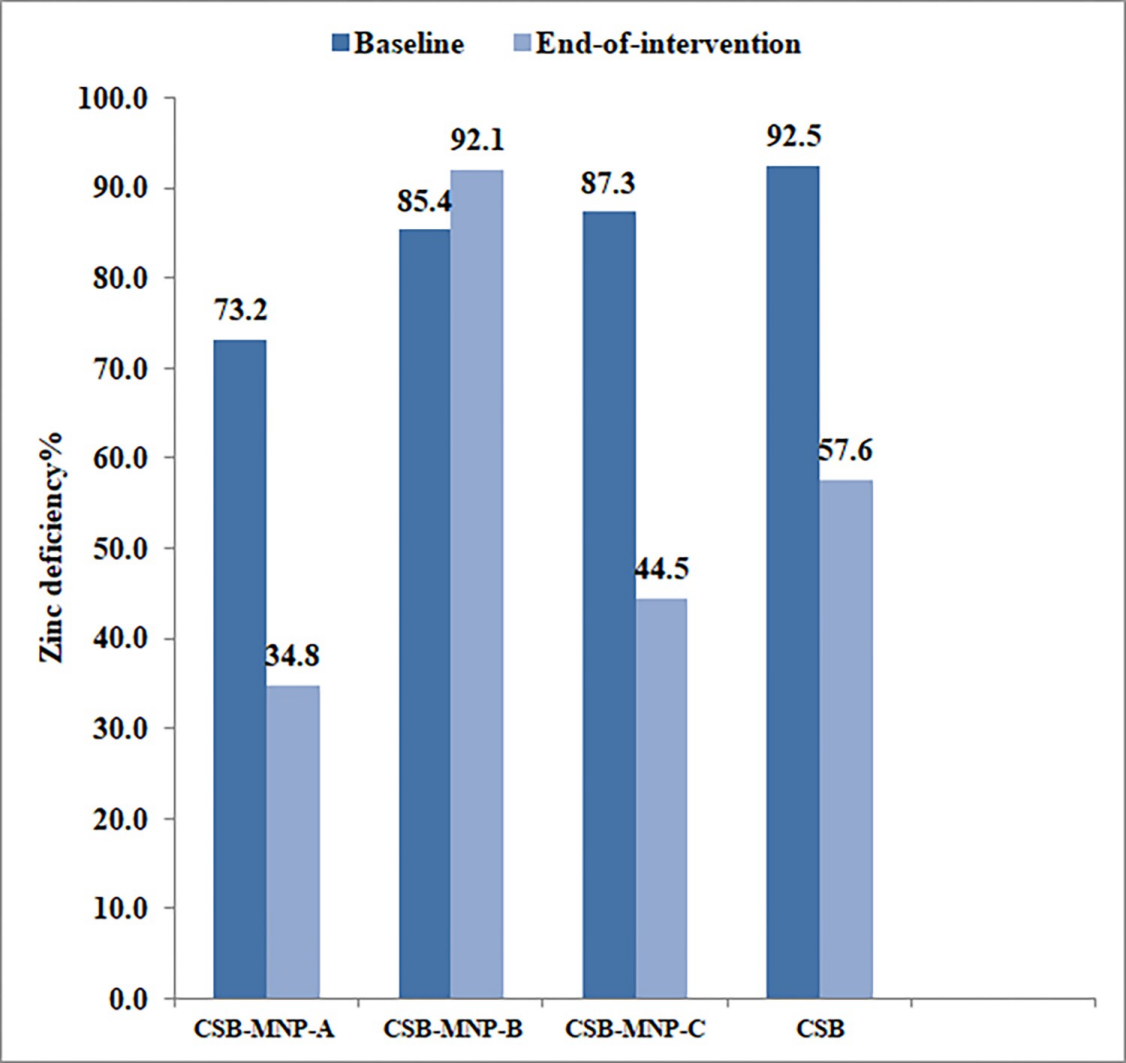
Zinc deficiency levels at baseline and end-of-intervention by study group.

The zinc deficiency levels of the study groups post-intervention are presented in Fig 2. Zinc deficiency decreased among children in the CSB-MNP-A CSB-MNP-C and CSB However, an increase in the proportion of zinc deficient children was observed in the CSB-MNP-B.

Bioequivalence fortified CSB to MNPs on the magnitude of change in serum zinc concentration were determined. The findings revealed that the standard CSB formulation was not equivalent to CSB-MNP-A, CSB-MNP-C and CSB-MNP-B within the ±20% bioequivalence limit (Table 4). The data is accessible in S1 File.

**Table 4:** Bioequivalence of MNPs to CSB on serum zinc concentration.

Bioequivalence to CSB (n = 80)	TOST^†^	T	p-value	Bioequivalence
CSB-MNP-C (n = 94)	Upper	0.27	0.317	
	Lower	-2.95	0.0254*	Non-quivalent
CSB-MNP-B (n = 88)	Upper	-5.15	0.996	
	Lower	-8.67	0.007**	Non-equivalent
CSB-MNP-A (n = 84)	Upper	2.73	0.028*	
	Lower	-0.68	0.572	Non-equivalent

Significant differences

*p <0.050

**p <0.010

^†^ TOST (Two one-sided t-tests)

**Fig 3:**
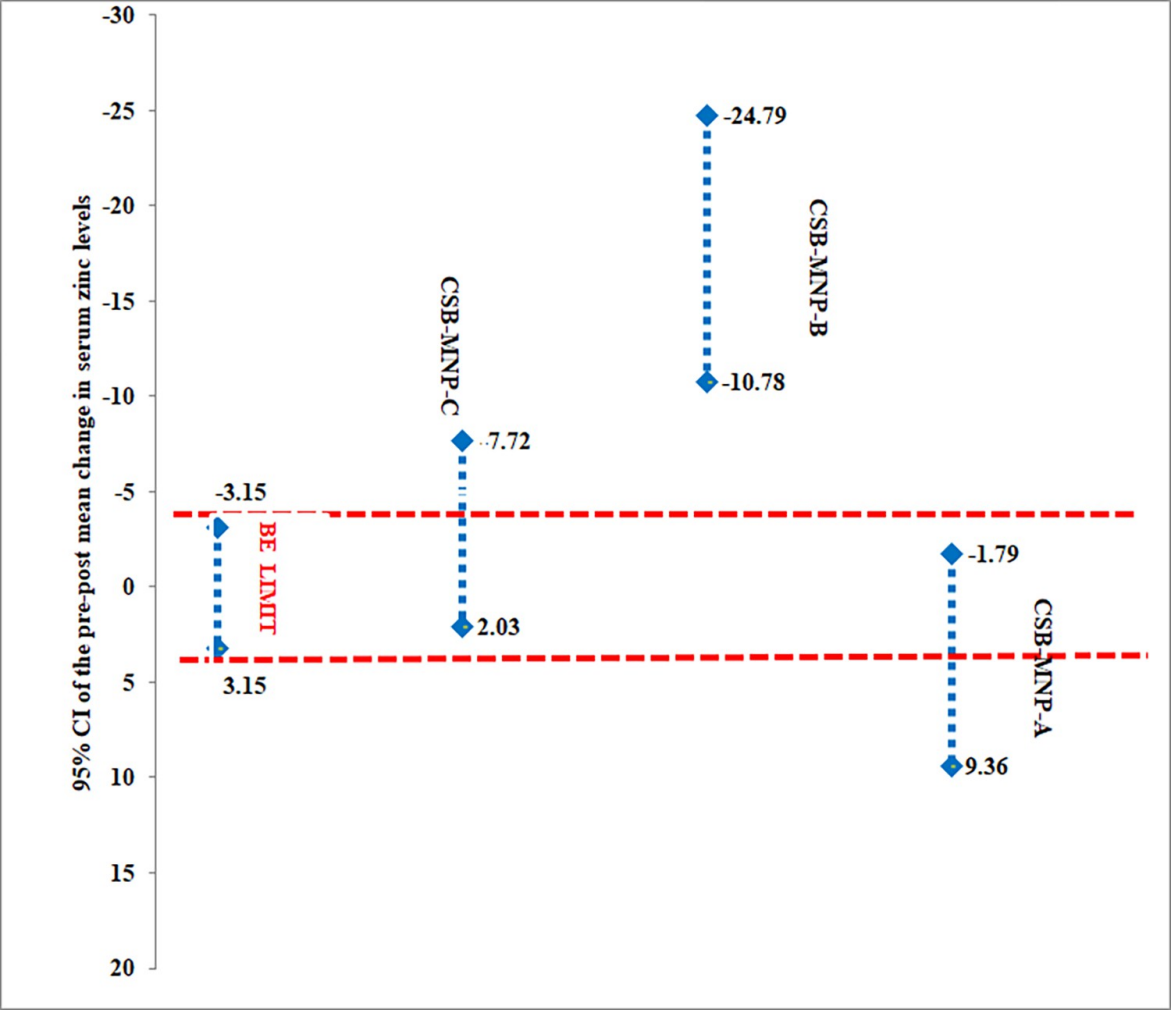
Bioequivalence of MNP formulations compared to CSB.

The confidence interval approach was also used to conclude lack of equivalence between the two treatments if one of the bounds of the 95% confidence interval for the geometric mean ratio of the outcomes for the treatment groups and comparator was found outside the bioequivalence limit. Fig 3 shows that the MNP formulations were not equivalent to CSB.

### Adverse effects

Mothers were advised to report any perceived adverse effects or undesirable responses by the child not limited to but including allergic response, diarrhoea and vomiting. A few (6) mothers reported diarrhoea after consumption of the porridge. However, it could not be ascertained whether the reported diarrhoea was as a result of the intervention, infections or due to other causes. One mother reported black stools which could be associated with iron fortification. Overall, there was no serious adverse events reported or observed which could have been attributed to the study.

## Discussion

To ensure adequate zinc nutriture, supplementary products for the treatment of children with MAM are usually fortified with zinc among other multiple micronutrient formulations. Serum zinc concentration has been shown to respond to zinc fortification interventions [33]. In the current study, there was an improvement in serum zinc concentration among children in study groups consuming zinc-fortified formulation (CSB-MNP-A, CSB-MNP-C and CSB). However, children in the CSB-MNP-B group who consumed a formulation which did not contain zinc showed lower serum zinc concentration compared to the baseline levels. This implies that there was a positive response of the children to zinc fortification of CSB irrespective of whether it was delivered in as the standard CSB or added through MNP fortification. The fact that majority of the children in the current study were initially zinc deficient could have contributed to the favourable response to zinc fortification. Enhanced absorption among zinc-deplete individuals has been reported [34, 35]. Multiple micronutrient interventions may have a greater potential to improve zinc status compared to single micronutrients as observed in our findings. This is possible owing to their complementary role. Other researchers have reported that vitamin A, zinc and iron interact with each other based on the absorption [36] and metabolism [37] thus affecting the bioavailability.

The findings of the current study further indicate that MNPs were not bioequivalent to CSB and that MNPs were more effective in improving the serum zinc concentration compared to CSB. The current findings imply that bioavailability of zinc in MNPs may be higher than CSB. The food matrix may have played a role in lowering the bioavailability of zinc in the CSB compared to MNPs. A review by Degerud and others reported that the bioavailability of zinc and other micronutrients when added to condiments and seasonings is affected by the food matrix [38]. Findings from a comparative study on fortification and supplementation of zinc indicate a potential of the food matrix affecting zinc absorption [39]. The fact that MNPs are added at the point of consumption may imply lesser food matrix interactions compared to fortified foods which have to undergo heat treatment during preparation. The food matrix effect observed in our study could have been due to the interaction of zinc with proteins during cooking resulting in cross-linkage of zinc and hence decreased bioavailability. Heating induces protein conformation changes in foods which may favor the cross-linkages. Potential protein-zinc cross-linkages have also been supported by the findings of an *in vitro* dializability study investigating the bioavailability of zinc and iron in cereals and pulses subjected to pressure cooking which showed a significant decrease in zinc bioavailability with a marked increase in iron bioavailability [40]. Further research is however needed to understand the possibility of the cross-linkage at the molecular level.

Bioequivalence testing in our study was conducted by comparing the pre-post change in serum zinc concentration for the experimental groups compared to the comparator group. However, it was also observed that improvement in zinc deficiency levels among the children receiving MNP multi-nutrient formulation containing zinc was better than those receiving CSB. It should be noted that the bioequivalence concept is commonly applied in pharmaceutical trials to establish the efficacy of generic drug compared to the branded counterparts with the same active ingredients. However, from the nutrition point of view, several factors may pose a challenge when evaluating bioequivalence. These factors range from physiological differences affecting bioavailability to the background dietary intakes of the study population [41], the later being subject to the limitations associated with the assessment methods. Likewise, the bioequivalence testing margins used for pharmaceutical products may not be comparable to that of nutrition products considering the public health importance of the interventions. Therefore, wider margins may be employed. Wider margins may favor bioequivalence of MNPs to CSB in the current study given that upper bioequivalence limit is likely to shift down. There is therefore need to develop a guideline for establishing bioequivalence for nutrition based products. The lack of equivalence between MNPs and CSB was however concluded. Ultimately, the choice of the fortification method may further be determined by other factors such as the target group, cost-effectiveness, ease of targeting the vulnerable group and sustainability. Trials designed considering larger sample sizes are however recommended to validate the current findings.

The strength of the current study is that the intervention was conducted in feeding centres where adherence to treatment was closely monitored. In addition to that, the bioequivalent trial was conducted among children with MAM who are likely to respond to food fortification due to existing micronutrient deficiencies. This guided the choice of the study population. Finally, the potential interaction of other micronutrients in the multiple micronutrient mix with zinc was assessed using positive controls (study groups consuming the formulation containing zinc only or multiple micronutrients mix without zinc. However, a limitation of the study is that the findings may not be generalized to populations that are not malnourished. Considering that in the management of children with MAM using fortified blended foods, MNPs are usually not recommended for safety reasons. We therefore recommend similar studies to be conducted to assess the bioequivalence of MNPs compared to CSB among normal/healthy children who are also prone to micronutrient deficiencies. There is also need for further research to assess the bioavailability of zinc in different food matrices while comparing the two methods of fortification.

### Conclusion

Fortification of CSB results to an increase in serum zinc concentration among MAM children. Our findings further demonstrate that MNPs are more effective in improving zinc status of children with MAM compared to CSB. However, reviewing the bioequivalence margin by considering the public health importance of nutrition interventions may favor bioequivalence of CSB to the MNPs. Trials with larger sample sizes are recommended to validate the current findings.

## Supporting information

S1 Checklist CONSORT 2010 checklist. (PDF)

S1 Protocol Study protocol. (PDF)

S1 File Data on bioequivalence of treatments. (XLS)

## References

[1] UNICEF. Malnutrition rates remain alarming: stunting is declining too slowly while wasting still impacts the lives of far too many young children. WHO and the World Bank Group Joint Child Malnutrition Estimates; 2020. Available from: *http://data.unicef.org/topic/nutrition/malnutrition/#*

[2] UNICEF. UNICEF Report Progress for Children–A Report Card on Nutrition, 2000–2006. 2007. Available from: https://www.unicef.org/publications/index_33685.html

[3] WHO. The WHO Child Growth Standards. (2006). Available from: http://www.who.int/childgrowth/publications/technical_report_pub/en/

[4] Jones KD, Thitiri J, Ngari M, Berkley JA. Childhood malnutrition: toward an understanding of infections, inflammation, and antimicrobials. Food Nutr. Bull. 2014; 35: 64–70. 10.1177/15648265140352S110 PMC4257992 25069296

[5] Concern Worldwide. Nutrition SMART Survey Conducted in the Slums of Nairobi County. May 2017. Available at http://www.nutritionhealth.or.ke/reports/smart-survey-reports/

[6] De Vita M, Scolfaro C., Santini B et al. Malnutrition, morbidity and infection in the informal settlements of Nairobi, Kenya: an epidemiological study. Ital J Pediatr. 2019; 45: 12. doi: 10.1186/s13052-019-0607-0 30642368 PMC6332593

[7] Olack B, Feikin DR, Cosmas LO, Odero KO, Okoth GO, Montgomery JM, et al. Mortality trends observed in population-based surveillance of an urban slum settlement, Kibera, Kenya, 2007–2010. PLoS ONE. 2014; 9(1). 10.1371/journal.pone.0085913 PMC3904840 24489678

[8] Bailey RL, West KP, Black RE. The Epidemiology of Global Micronutrient Deficiencies. Ann Nutr Metab 2015; 66 (suppl 2): 22–33. doi: 10.1159/000371618 26045325

[9] Abubakar N, Atiku MK, Alhassan AJ, Mohammed IY, Garba RM, Gwarzo GD. An assessment of micronutrient deficiency: A comparative study of children with protein-energy malnutrition and apparently healthy controls in Kano, Northern Nigeria. Trop. J. Med. Res. 2017; 20: 61–5

[10] Kenya Ministry of Health. Kenya National Micronutrient Survey 2011; 2011.

[11] Black RE. Global distribution and disease burden related to micronutrient deficiencies. *Nestlé Nutrition Institute Workshop Series*, 2014; 78: 21–28. doi: 10.1159/000354932 24504203

[12] Biesalski HK, Black RE (eds). Hidden Hunger. Malnutrition and the First 1,000 Days of Life: Causes, Consequences and Solutions. World Rev Nutr Diet. Basel, Karger. 2016; 115: 125–133 10.1159/000442079

[13] Guerrant RL, DeBoer MD, Moore SR, Scharf RJ, Lima AA. The impoverished gut—a triple burden of diarrhoea, stunting and chronic disease. Nat Rev Gastroenterol Hepatol. 2012; 10(4): 220–229. doi: 10.1038/nrgastro.2012.239 23229327 PMC3617052

[14] Ackland ML, Michalczyk AA. Zinc and infant nutrition. Arch. Biochem biophys. 2016; 611:51–57. doi: 10.1016/j.abb.2016.06.011 27317042

[15] Macharia-Mutie CW, Brouwer ID, Mwangi AM, Kok FJ. Complementary feeding practices and dietary intake among children 12–23 months in Mwingi district, Kenya. Int J Food Saf Nutr Publ Health. 2010; 3(1): 45. 10.1504/IJFSNPH.2010.032034

[16] Abolurin OO, Oyelami OA, Oseni SB. A comparative study of the prevalence of zinc deficiency among children with acute diarrhoea in South Western Nigeria. Afri Health Sci. 2020; 20(1): 406–12. 10.4314/ahs.v20i1.47 PMC7750047 33402929

[17] Wessells KR, Brown KH. Estimating the global prevalence of zinc deficiency: results based on zinc availability in national food supplies and the prevalence of stunting. PLoS ONE. 2012; 7(11), e50568. doi: 10.1371/journal.pone.0050568 23209782 PMC3510072

[18] Rosalind SG, Victor R, Janet CK. Implications of phytate in plant-based foods for iron and zinc bioavailability, setting dietary requirements, and formulating programs and policies. Nutr. Rev. 2018; 76 (11): 793–804. doi: 10.1093/nutrit/nuy028 30010865

[19] De Pee S, Bloem M. Current and potential role of specially formulated foods and food supplements for preventing malnutrition among 6–23 months old and treating moderate malnutrition among 6–59 months old children. Food Nutr. Bull. 2009; 30: 434–463. 10.1177/15648265090303S305 19998866

[20] Bhutta ZA, Hurrell RF, Rosenberg IH (eds). Meeting Micronutrient Requirements for Health and Development. Nestlé Nutr Inst Workshop Ser Nestec Ltd., Vevey/S. Karger AG., Basel. 2012; 70: 11–21. 10.1159/000337388

[21] Roos N, Sørensen JC, Sørensen H, Rasmussen SK, Briend A, Yang Z, et al. Screening for anti-nutritional compounds in complementary foods and food aid products for infants and young children. Matern Child Nutr. 2013; 9 Suppl 1(Suppl 1): 47–71. doi: 10.1111/j.1740-8709.2012.00449.x ; PMCID: PMC6860611. 23167584 PMC6860611

[22] Hurrell R, Egli I. Iron bioavailability and dietary reference values. Am. J. Clin. Nutr. 2010; 91(5): 1461S–1467S. doi: 10.3945/ajcn.2010.28674F 20200263

[23] Chege PM, Ndungu ZW, Gitonga BM. Food security and nutritional status of children under-five in households affected by HIV and AIDS in Kiandutu informal settlement, Kiambu County, Kenya. J Health Popul Nutr. 2016; 35(1): 21. doi: 10.1186/s41043-016-0058-9 27443524 PMC5025998

[24] Chow SC, Liu JP. Design and analysis of bioavailability and bioequivalence studies. Marcel Dekker, New York; 1992.

[25] Cohen J. Statistical power analysis for the behavioral sciences. 2nd ed. Hillsdale, NJ: Lawrence Earlbaum Associates; 1988.

[26] Kenya Demographic and Health Survey. National Council for Population and Development. Nairobi: Government printers; 2014.

[27] IZiNCG. Assessing population zinc status with serum zinc concentration. Technical Brief No. 2; 2007. Available from: https://www.izincg.org/technical-briefs

[28] Namaste SML, Aaron GJ, Varadhan R, Peerson JM, Suchdev PS; BRINDA Working Group. Methodologic approach for the Biomarkers Reflecting Inflammation and Nutritional Determinants of Anemia (BRINDA) project. Am J Clin Nutr. 2017; 106 (Suppl):333S–47S. doi: 10.3945/ajcn.116.142273 28615254 PMC5490643

[29] Doumas BT, Watson WA, Biggs HG. Albumin standards and the measurement of serum albumin with bromocresol green. Clin Chim Acta 1971; 31: 87–96. 5544065 10.1016/0009-8981(71)90365-2

[30] Rozga J, Piątek T, Małkowski P. Human albumin: old, new, and emerging applications. Ann. Transpl. 2013; 18: 205–217. 10.12659/AOT.889188 23792522

[31] Piaggio G, Carroli G, Villar J, Pinol A, Bakketeig L, Lumbiganon P, et al. Methodological considerations on the design and analysis of an equivalence stratified cluster randomization trial. Stat Med. 2001; 20(3):401–16. doi: 10.1002/1097-0258(20010215)20:3&lt;401::aid-sim801&gt;3.0.co;2-1 11180310

[32] Ip H, Hyder SM, Haseen F, Rahman M, Zlotkin SH. Improved adherence and anaemia cure rates with flexible administration of micronutrient Sprinkles: a new public health approach to anaemia control. Eur J Clin Nutr. 2009; 63: 165–172. doi: 10.1038/sj.ejcn.1602917 17895911

[33] Das JK, Salam RA, Kumar R, Bhutta ZA. Micronutrient fortification of food and its impact on woman and child health: a systematic review. Syst. Rev. 2013; 2(1): 67. doi: 10.1186/2046-4053-2-67 23971426 PMC3765883

[34] Roohani N, Hurrell R, Kelishadi R, Schulin R. Zinc and its importance for human health: An integrative review. J Res Med Sci. 2013; 18(2):144–157. 23914218 PMC3724376

[35] Krebs NF. Overview of zinc absorption and excretion in the human gastrointestinal tract. J. Nutr. 2000; 130(5): 1374S–1377S. doi: 10.1093/jn/130.5.1374S 10801946

[36] Dekker LH, Villamor E. Zinc supplementation in children is not associated with decreases in haemoglobin concentrations. J. Nutr. 2010; 140(5): 1035–1040. 10.3945/jn.109.119305 20335624

[37] Muñoz EC, Rosado JL, López P, Furr HC, Allen LH. Iron and zinc supplementation improves indicators of vitamin A status of Mexican preschoolers. Am. J. Clin. Nutr. 2000; 71(3): 789–794. doi: 10.1093/ajcn/71.3.789 10702174

[38] Degerud E, Manger M, Strand T, Dierkes J. Bioavailability of iron, vitamin A, zinc, and folic acid when added to condiments and seasonings. Ann Ny Acad Sci. 2015; 1357 (1). doi: 10.1111/nyas.12947 26469774 PMC5019242

[39] Brown KH, de Romaña DL, Arsenault JE, Peerson JM, Penny ME. Comparison of the effects of zinc delivered in a fortified food or a liquid supplement on the growth, morbidity, and plasma zinc concentrations of young Peruvian children. Am. J. Clin. Nutr. 2007; 85 (2): 538–547. doi: 10.1093/ajcn/85.2.538 17284755

[40] Yuwei L, Zhenping H, Qian W. Impact of heat processing on the bioavailability of zinc and iron from cereals and pulses. Int. Food Res. J. 2017; 24(5):1980–1985.

[41] Lichtenstein AH, Yetley EA, Lau J. Application of Systematic Review Methodology to the Field of Nutrition: Nutritional Research Series, Vol. 1. Rockville (MD): Agency for Healthcare Research and Quality (US); 2009. Technical Reviews, No. 17.1. Available from: https://www.ncbi.nlm.nih.gov/books/NBK44076/20734513

