# Supplementary material for: Bioequivalence of micronutrient powders to Corn-soy Blend on serum zinc concentration of children (6–36 months) with Moderate Acute Malnutrition in Thika urban slums, Kenya: A cluster-randomized controlled trial
Source: PLoS One. 2022 Sep 19;17(9):e0274870. doi: 10.1371/journal.pone.0274870 (PMC9484639; doi:10.1371/journal.pone.0274870)
Supplement: S1 Protocol — (PDF) [file pone.0274870.s002.pdf]

STUDY PROTOCOL

Study design

The study will adopt a cluster randomized bioequivalence trial with four study groups; zinc, zinc in multiple micronutrients and multiple micronutrients without zinc (experimental groups adding once daily Micronutrient Powders (sprinkles^{tm}) to corn-soy porridge) and a comparator group on daily conventionally fortified Corn-Soy Blend (CSB).

Study area

The study will be based in Kiandutu and adjacent informal settlements, 40 km North of Nairobi, situated in Thika West District, Kiambu County, approximately 40 km north of the City of Nairobi. The district lies between latitudes 3°53' and 1° 45' South of Equator, longitudes 36° 35' and 37° 25' East and altitude 1631m above sea level. Kiandutu slum is a high density informal settlement with an estimated population of over 70,000 (2009 census report) and is one of the largest informal settlements located outside Nairobi, the capital city of Kenya. Kiandutu means a place of "jiggers" depicting poor living conditions (Koomen, 2012). The collapse of industries and coffee plantations in Thika District rendered most of the inhabitants jobless and hence the high poverty levels (Kinyanjui, 2007).

The study area will consist of twelve villages; Mtatu, Biashara, Muslim, Kianjau, Molo, Mkira, Wahome, Athena settlement, Makongeni, Umoja, Gachagi and Madharau. Two public health facilities are located in the study area; Kiandutu Health Centre (HC) and Makongeni Health Centre and are supported up-stream by Thika Level Five Hospital where referrals are made for the critically ill individuals. The study area was selected because research has shown that children living in informal settlements have a high prevalence of malnutrition aggravated by poverty, food insecurity, poor hygiene and sanitation and limited access to health services.

Baseline study

At baseline, information on socio-economic status, vaccination status, appetite, morbidity patterns, dietary patterns, anthropometric measurements, serum zinc(zn), serum albumin and CRP of the children will be collected.

Intervention phase

Target population

The study will target Moderately Malnourished (MM) children 6-36 months old with height weight for height (WH) within -3 and less than -2 Z-scores or with/and a mid-upper arm circumference (MUAC) within the range of 115-125 mm without bilateral oedema. The age 6-36 months is a period of rapid growth, highest nutrient requirements and poor diets pre-dispose children to a risk of zinc deficiency. The beneficial effect of zinc may be limited to children with pre-existing growth failure (IZiNCG, 2004b) hence the study targets MM children. The respondents will be the mothers/primary caregivers to the children.

Inclusion criteria

MM children (6-36) months, whose parents consent, willing to be visited in their homes and intending to stay in the area during the study period and who will have stayed in the area for the past 6 months will be recruited.

Exclusion criteria

The study will exclude severely malnutrition and bed-ridden children. Those with other conditions as verified from medical cards, review by a clinical officer, interview sessions and observations will also be excluded. Children taking micronutrient supplements (except Vit. A) and/or fortified foods for the past 3 months will also be excluded. This information will be verified from medical cards and recording of the supplements or fortified foods given to the child as indicated in the food labels.

Description of the intervention

CSB (80% corn and 20% soy) and sprinkles meeting 100% RDA of zinc will be sourced from suppliers. For a period of six months, sprinkles (1g) will be added to corn-soy porridge once daily and CSB porridges made from 100g flour will be given daily (Figure 1). The micronutrient composition of the different study groups is shown in Table 1. The food supplements will be prepared in feeding centres. De-worming will be done at the start of the intervention using mebendazole (500 mg). Demonstrations on porridge preparation and use of sprinkle and nutrition education focused on the importance of nutritious diet will be given to the primary caregivers on group basis at baseline.

Table 1: Micronutrient composition of sprinkles and CSB

	Expt Gp 1: Sprinkles (per l g sachet)	Expt Gp 2: Sprinkles* (per l g sachet)	Expt Gp 3: Sprinkles* (per l g sachet)	Comparator Gp 4: CSB (l00g dry wt.)
Vit A (IU)	-	1250	1250	1300
Vit E (mg)	-	6.0	6.0	-
Vit C (mg)	-	30	30	30
Thiamine (mg)	-	0.5	0.5	0.1
Riboflavin (mg)	-	0.5	0.5	0.4
Vit B6 (mcg)	-	0.5	0.5	-
Niacin (mg)	-	6	6	5
Folic Acid (mcg)	-	160	160	50
Vit. B12 (mcg)	-	0.9	0.9	1
Vitamin D3	-	5	5	-
Calcium (mg)	-	-	-	100
Iron (mg)	-	12.5	12.5	8
Zinc (mg)	5	5	-	5
Iodine (mcg)	-	90	90	-
Copper	-	0.3	0.3	-

Randomization

The sampling frame will consist of the 12 villages in Kiandutu slum. Three villages will be randomly allocated to each of the 4 study groups. Villages rather than study subjects will be randomized to avoid cross-contamination of information due of the close proximity of the study subjects. Computer generated randomization will be done by a statistician.

Data collection tools and procedures

Baseline data on children's past and present illnesses will be obtained by interviewing the primary caregivers and from confirmation from health cards. A semi-structured questionnaire designed to elicit information on the household socio-economic, demographic structure, parental education and employment, material possessions and type of housing materials. A 24-hour recall designed for measuring nutrient intakes inclusive of zinc will be used and a Food Frequency Questionnaire (FFQ) will be administered to cover the most commonly consumed complementary foods based on a 7-day recall. Appetite will be assessed monthly using the Children's Eating Behaviour Questionnaire (CEBQ),

Serum zn, albumin and CRP will be determined at baseline and post-intervention. Monthly anthropometric measurements and dietary intake will be conducted monthly for the 6-month period. Interviewers will visit the childrens' homes bi-weekly to inquire about symptoms of illness (severity, frequency and duration) of diarrhoea, ARTI and other infections, and monitor the feeding practices.

Training of research team

A research team comprising of a clinical officer, and 6 Research Assistants (RA's) (minimum education; K.C.S.E) and interviewers will be trained on the research aspects. The objectives will be explained without disclosing the research hypotheses. Interviewers will be trained on taking anthropometric measurements and interviewing skills taught through demonstrations and role plays. The investigator will test the RA's and interviewers for accuracy, consistency and logical recording skills, and corrective measures taken till the investigator was satisfied with the competence.

3.4.6 Sample size determination

Determination of sample size will be based on the primary objective and the formula by Liu & Chow (1992). Where; $n = \dfrac{(Z_\alpha + Z_\beta)^2 \sigma^2}{(\delta - |\varepsilon|)^2} = \dfrac{(1.64 + 1.28)^2 0.71^2}{(0.2 - 1.0)^2} = 67$

Where; n = No. of study subjects per group, σ= variance within the groups, δ = expected outcome difference of the groups (20 % as per the bioequivalence rule), ε = the absolute values of proportion differences among the groups. The standard normal deviate at the required confidence interval (95 probability error).

The prevalence of zinc deficiency in Kenya is 50% (GOK & UNICEF, 2008). The estimated post-intervention prevalence of 20%, 10%, 20% and 50% for the reference, ZN, ZN in MMN and MMN without ZN groups respectively. To show equivalence within 20% rule with 80% power and 5% significance, a sample size of 67 per group was obtained and allowing 20% attrition, a sample size of 80 per group is obtained.

Where; n = The desired sample size, z = The standard normal deviate at the required confidence interval (95 probability error equal to 1.96), p =The proportion of occurrence of the phenomenon (0.5) since prevalence is 50%), q= The proportion of non-occurrence is (1-P) = 0.5, d = level of statistical significance (0.05 for 95% level of significance).

Recruitment of study participants

Door-to-door visits will be done to identify families with the target children. The children will then be screened by a clinical officer. The consent form and procedures of the intervention will be explained to the parents of the eligible children and a thumb print or written informed consent obtained from the interested parents. A follow up will be done within 2 days to start the intervention.

Pilot study

A Pilot study will be conducted (in Kiangombe slums, adjacent to Kiandutu slums and with similar socio-economic and demographic characteristics) on 10 % of the targeted sample size for each study group to optimize the study procedures, pre-test instruments and to provide information on other factors so as to aid in the planning of the main study.

Validity and reliability

Questionnaires will be pre-tested in the pilot study to ascertain their reliability and validity. The Socio-demographic Questionnaire, FFQ and CEBQ used in this study have been validated (KDHS, 2008-09, Gibson & Ferguson, 2008, Wardle *et al.*, 2007).

Anthropometric measurements

Weight will be measured on a pediatric balance-beam scale to the nearest 0.1 kg while length/height to the nearest 0.1 cm taken when nude. Recumbent length will be taken for children less than two years or those whose length is shorter than 87cm and height for those whose length is greater than 87 cm. Age as per birth date (Child Health Cards) will be taken. When such evidence misses, age will be estimated using the parent's estimates or local event calendars. Oedema will be confirmed if a shallow print remains on both feet, after applying thumb pressure for 30 seconds to the two feet simultaneously. ENA for SMART software will be used to standardize anthropometric measurements.

Determinations of hair and serum zinc status, serum albumin and CRP levels

Fasting morning blood (5 ml) will be collected from the children by venipuncture by a medical technician from KEMRI. After centrifuging (750 x *g*) for 10 min, aliquots of serum will be transferred into trace elements free tubes and put in a cool box containing ice pack and frozen later the same day at –20°C (AOAC, 1995). Hair samples <0.15g will be collected from the occipital region of the scalp by using stainless steel scissors**.** The proximal 1–2cm of

hair will be washed and wet digested for with nitric acid. Serum and hair zinc levels will be determined by Atomic Absorption Spectrometry.

Serum albumin will be determined by HPLC (Rodkey, 1965). Serum will be diluted with a solution of bromcresol green and absorbance measurements made at 615 μm. CRP will be analyzed using ELISA method. Microwells will be coated with 100µl/well of rabbit anti-human CRP diluted 1:88 in coating buffer and incubated at 4ºC overnight. Wells will be washed and 100µl/well of serum samples added and diluted 1:8,000 in diluting buffer. After incubation for 2 hours, and washing, peroxidase-conjugated rabbit antihuman diluted at 1:6,000 in diluting buffer added to the well at 100µl/well and incubated for 1 hour. The plates will be washed and the chromogen added at 100µl/well. After incubation for 15 minutes in the dark, the reaction will be stopped with 100μl/well of 0.5M H_2S0_4. And the plates will be read at 490nm.

Data analyses

The field data will be cleaned and coded and analyzed using SPSS software. Socio-economic (SE) status data will be reduced by using principal components analyses to an index using STATA. ENA for SMART will be used to analyse anthropometric data. The indices used to determine nutritional status will be; WA, HA and WH. Nutritional status will be judged by the WHO standards. Child feeding practices will be assessed using the indicators as per WHO (2001b) and WHO (2007) IYCF guidelines. Child feeding index, breastfeeding and complementary feeding (initiation, meal frequency and dietary diversity as a proxy for quality) will be determined. Nutrient intakes will be analyzed using Nutri-survey. Relative risk and odds ratio will be used to show the association between morbidity patterns and zinc status. Pearson product moment correlation and chi-square will be used to determine the relationship between non-categorical variables (feeding practices and nutrition status, SE status) and categorical variables (sex, age, type of illness and maternal characteristics) with zinc status respectively.

The relationships between the categorical variables will be analyzed to assess inter-group differences in dietary practices, nutrition status, morbidity patterns and serum and hair zn status at baseline, monthly and end of the study using chi-square and t-test, ANOVA and ANCOVA. Simple linear regression will be used to determine the contribution of sprinkles/CSB, dietary practices and SE status to nutrition status, morbidity patterns and

serum zn levels. Multiple regression analysis will be used to determine the extent to which sprinkles/fortification, dietary practices and predict nutrition status, morbidity patterns and serum zn levels and logistical regression used to analyze for confounding variables. Content analysis will be used for qualitative data from FGDs by summarizing the information into emerging themes. Bioequivalence of sprinkles to CSB will be drawn if the percentages of children with serum zn <9.9 μmol/L is within ± 20 rule (excluding CRP levels >10 mg/l), the basis to accept or reject the null hypothesis. Significance will be determined at 95% confidence interval, P-value of 0.05.

Logistical and ethical considerations

Clearance will be sought from the Graduate School, Kenyatta University and a research permit obtained from the Ministry of Education. Authority to conduct research will be obtained from the Ministry of Health, Thika and the local chief. Ethical approval will be obtained from the KEMRI National Ethical Review Committee and a thumb print or written informed consent sought from the parents, after explaining the purpose of the study without revealing the study hypotheses. The food supplements will also be given to all the underfive children in the intervention households. Sick children will be advised to seek further medical attention at Thika Level 5 Hospital. Blood will be drawn two times during the study from children whose parents consent. The information provided will be treated in the strictest confidence and none of the participants will be individually identifiable in the resulting thesis and that it will be used for the purpose of the study only. The outcome of research will be communicated to the respondents.
